# Exploring the Prognostic Role of Ki67 Proliferative Index in Merkel Cell Carcinoma of the Skin: Clinico-Pathologic Analysis of 84 Cases and Review of the Literature

**DOI:** 10.1007/s12022-020-09640-3

**Published:** 2020-07-22

**Authors:** Stefano La Rosa, Matteo Bonzini, Amedeo Sciarra, Sofia Asioli, Roberta Maragliano, Martina Arrigo, Maria Pia Foschini, Alberto Righi, Francesca Maletta, Alberico Motolese, Mauro Papotti, Fausto Sessa, Silvia Uccella

**Affiliations:** 1 Institute of Pathology, University Hospital and University of Lausanne, Lausanne, Switzerland; 2 Institut Universitaire de Pathologie, CHUV, 25 rue du Bugnon, CH-1011 Lausanne, Switzerland; 3 Department of Clinical Sciences and Community Health, University of Milan and IRCCS Policlinico Maggiore Hospital Foundation, Milan, Italy; 4 Division of Pathology, University of Milan and IRCCS Policlinico Maggiore Hospital Foundation, Milan, Italy. Current affiliation: Department of Histopathology, Central Institute, Valais Hospital, Sion, Switzerland; 5 Unit of Pathology, Bellaria Hospital and Department of Biomedical and Neuromotor Sciences, University of Bologna, Bologna, Italy; 6 Department of Medicine and Surgery, University of Insubria, Varese, Italy; 7 Dermatology Unit, ASST Sette Laghi, Varese, Italy; 8 Department of Pathology, IRCCS Istituto Ortopedico Rizzoli, Bologna, Italy; 9 Department of Oncology, City of Health and Science, University of Turin, Torino, Italy

**Keywords:** Merkel cell carcinoma, Ki67 standardized count, Prognosis, Overall survival, Disease-specific survival

## Abstract

The exact prediction of outcome of patients with Merkel cell carcinoma (MCC) of the skin is difficult to determine, although several attempts have been made to identify clinico-pathologic prognostic factors. The Ki67 proliferative index is a well-known marker routinely used to define the prognosis of patients with neuroendocrine neoplasms. However, its prognostic value has been poorly investigated in MCC, and available published results are often contradictory mainly because restricted to small series in the absence of standardized methods for Ki67 evaluation. For this reason, we explored the potential prognostic role of Ki67 proliferative index in a large series of MCCs using the WHO standardized method of counting positive cells in at least 500 tumor cells in hot spot areas on camera-captured printed images. In addition, since MCC may be considered as the cutaneous counterpart of digestive neuroendocrine carcinomas (NECs), we decided to stratify MCCs using the available and efficient Ki67 threshold of 55%, which was found prognostic in digestive NECs. This choice was also supported by the Youden index analysis. In addition, we analyzed the prognostic value of other clinico-pathologic parameters using both univariate and multivariate analysis. Ki67 index appeared significantly associated with prognosis at univariate analysis together with stage IV, lack of MCPyV, and p63 expression, but not at the multivariate analysis, where survival resulted independently influenced by p63 expression and tumor stage, only.

## Introduction

Merkel cell carcinoma (MCC) is a rare neuroendocrine carcinoma of the skin, but its incidence has largely increased over the last 20 years, probably depending on the increasing effects of risk factors such as advanced age, UV exposure, and systemic immunosuppression [1]. The reported annual incidence varies between 0.1 and 0.88 per 100,000 people, with differences among geographical regions: higher rates have been observed in Australia and New Zealand and the lowest in Eastern France and Scotland [2]. MCC is more frequently observed in elderly white male patients (mean age at diagnosis of 73.6 years), although younger patients, mostly if immunosuppressed due to organ transplantation, can also be affected. MCC incidence is also increased in patients with other malignancies including melanoma, multiple myeloma, and non-Hodgkin lymphoma, especially chronic lymphocytic leukemia [2].

MCC is an aggressive cancer, and several attempts have been made to identify clinico-pathologic factors useful to stratify patients in different prognostic categories. From an etiological point of view, MCC can be divided into two main groups with apparent different behaviors on the basis of the presence or absence of an associated infection by Merkel cell polyomavirus (MCPyV). MCC unassociated with MCPyV infection seems to show a worse prognosis [3]. However, although several prognostic markers have been proposed, the exact prediction of the individual outcome remains difficult to be determined.

Ki67 proliferative index is a well-known prognostic marker for both well-differentiated neuroendocrine tumors (NETs) and poorly differentiated neuroendocrine carcinomas (NECs) of the digestive system, and is currently routinely evaluated in their diagnostic work-up [4]. Although its prognostic value has also been recognized in other neuroendocrine neoplasms (NENs), such as those located in the pituitary, parathyroid, and lung [5–8], Ki67 proliferative index has been poorly investigated in cutaneous MCC; thus, its role remains to be clarified.

In the present retrospective study, we explored the prognostic role of Ki67 proliferative index in a large series of MCCs integrating our results with literature findings. In addition, we investigated other potential clinico-pathologic prognosticators, with the aim to identify useful parameters to stratify MCC patients in different risk categories.

## Materials and Methods

### Cases

The surgical pathology databases of the Units of Pathology of the ASST Sette Laghi/University of Insubria (Varese, Italy), of the Bellaria Hospital/University of Bologna (Bologna, Italy), of the Istituto Ortopedico Rizzoli (Bologna, Italy), and of Città della Salute e della Scienza/University of Turin (Turin, Italy) were retrospectively analyzed to identify cutaneous MCCs diagnosed between 1993 and 2015.

The clinico-pathological information including gender, age at the time of diagnosis, tumor site and size, presence of lymph node and/or distant metastases, stage, and available clinical follow-up data were collected from hospital medical records, from general physician or referring specialist, and from local Tumor Registry. Patients were followed up for at least 36 months after surgery.

### Morphological and Immunohistochemical Analysis

Tissues were fixed in buffered formalin and routinely processed to paraffin. Histological slides were stained with hematoxylin and eosin and reviewed by three pathologists with an expertise in neuroendocrine neoplasms (SA, SLR, and SU) to confirm the diagnosis and to evaluate the following morphological parameters: diameter, thickness of infiltration, mitotic count (number of mitoses per 2 mm^2^), angioinvasion, and margin status. In general, immunohistochemical stains for synaptophysin, chromogranin A, and cytokeratin 20 were already available because routinely performed in the diagnostic workup. The other immunohistochemical markers, including Ki67, p63, and MCPyV, were performed when missing in the original records. Immunohistochemistry was performed in an automated stainer (Benchmark XT; Ventana Medical Systems, Tucson, AZ) using 3-μm-thick sections and the antibodies listed in Table 1. Ki67 proliferative index was evaluated in one selected block and always at distance from ulceration, if present. The distribution of Ki67 labelling was rather homogeneous in cases with the highest scores (> 55%), while it was heterogeneous when the percentage of positive cells was lower. In agreement with the most recent guidelines, Ki67 proliferative index in case with heterogeneous Ki67 expression was evaluated in the hot spot area (selected at low magnification) by counting the number of positive cells in at least 500 tumor cells (range 500–2000 neoplastic cells) on camera-captured printed images [4, 9].

**Table 1:** Antibodies and antisera used

Antibody	Dilution	P/M (clone)	Source
Synaptophysin	1:100	M (snp88)	BioGenex Laboratories, San Ramon, CA, USA
Chromogranin A	1:1	M (LK2H10)	Ventana Medical System, Tucson, AZ, USA
CK20	1:100	M (K_5_20.8)	Dako Corporation, Carpinteria, CA, USA
p63	1:2	M (4A4)	Cell Marque, Roklin, CA, USA
Ki67	1:100	M (MIB1)	Dako
MCPyV	1:100	M (CM2B4)	Santa Cruz Biotechnology Inc., Santa Cruz, CA, USA

*P/M*, polyclonal/monoclonal; *CK*, cytokeratin; *MCPyV*, Merkel cell polyomavirus

### Statistical and Survival Analysis

Summary statistics have been reported as number and percentage, mean (± standard deviation-SD) or median (standard error (SE), and corresponding 95% confidence intervals (CI)) when appropriate. Student’s *t* test has been performed to compare continuous variable across groups. Chi square tests were used to compare percentage thresholds across groups. All possible prognostic factors were submitted to a Cox proportional-hazard regression. All factors with a *p* level ≤ 0.05 then entered in a multivariate Cox regression, along with potential confounders. Hazard ratios (HR) and 95% CI are also indicated. Kaplan–Meier estimator was used to estimate the survival function from lifetime data including overall survival and disease-specific survival cumulative rates. Formal test for equality of survivor function was performed by log rank test. Optimal cut-point for Ki67 index was investigated using Youden’s index for the receiver operating characteristic (ROC) curve analysis. A *p* value < 0.05 was considered significant. All tests were two-sided. Analyses were performed with Stata14.2 (®2013 Stata Corp Austin, US) and SPSS 26.0 (®2019 SPSS Inc., Chicago, IL, USA).

### Revision of the Literature

The PubMed database of the National Center for Biotechnology Information (NCBI) of the US National Library of Medicine was searched using the following string: Ki67 [AND] Merkel cell carcinoma or proliferation [AND] Merkel cell carcinoma. All articles written in English were included. In addition, we revised the reference lists of each paper selected in the PubMed database, with the aim of reducing the risk of missing pertinent articles. For each identified article, the following information was considered: number of cases in each series, the mean Ki67 proliferative index value, the Ki67 cut-off selected for survival analyses, and correlation of the Ki67 proliferative index with disease recurrence, overall (OS), and disease-specific (DSS) survival.

## Results

### Clinico-Pathological and Immunohistochemical Results

From a larger series of 100 patients, 84 cases had complete clinical information and material for immunohistochemical characterization available. The main clinico-pathologic features of the 84 patients are summarized in Table 2. Males were more frequently affected than females (*p*: 0.02), and the average age at diagnosis was 76 years (range 42–94 years). Mean age was slightly higher among women (79 vs 74 years).

**Table 2:** Main clinico-pathological features of Merkel cell carcinomas

Gender	
Men	50/84 (60%)
Women	34/84 (40%)
Age (years)	
Mean	76
Range	42–94
Site	
Head and neck	31
Trunk and buttock	17
Extremities (3)	36
Tumor diameter (cm)	
Mean	2.49
Range	0.3–12
Tumor thickness (mm)	
Mean	9.24
Range	0.5–21
Angioinvasion	
Yes	54/84 (64%)
No	30/84 (36%)
Mitoses × 2mm^2^	
Mean	37
Range	4–180
Ki67 ≥ 55%	30/84 (36%)
Ki67 < 55%	54/84 (64%)
Stage I	27/84 (32%)
II	23/84 (27%)
III	23/84 (27%)
IV	11 /84 (13%)
p63 +	38/84 (45%)
p63 −	46/84 (55%)
MCPyV +	71/84 (85%)
MCPyV −	13/84 (15%)

*MCPyV* Merkel cell polyomavirus

The mean diameter of MCCs was 2.49 cm (range 0.3–12), and the mean thickness of infiltration was 9.24 mm (range 0.5–21 mm). All tumors showed the classical morphological features of skin MCC including small to intermediate cells with monomorphic nuclei displaying a finely dispersed chromatin, inconspicuous nucleoli, and scant cytoplasm. Co-existent non-neuroendocrine components (including squamous cell carcinoma or basal cell carcinoma) were never observed. The mean mitotic count was 37 mitoses × 2mm^2^ and 54 cases (64%) showed angioinvasion. MCPyV nuclear immunoreactivity was identified in 71 cases (85%) and p63 expression in 38 cases (45%). The mean Ki67 proliferative index was 51.3%, ranging between 20 and 95%.

### Survival Analysis

**Fig. 1:**
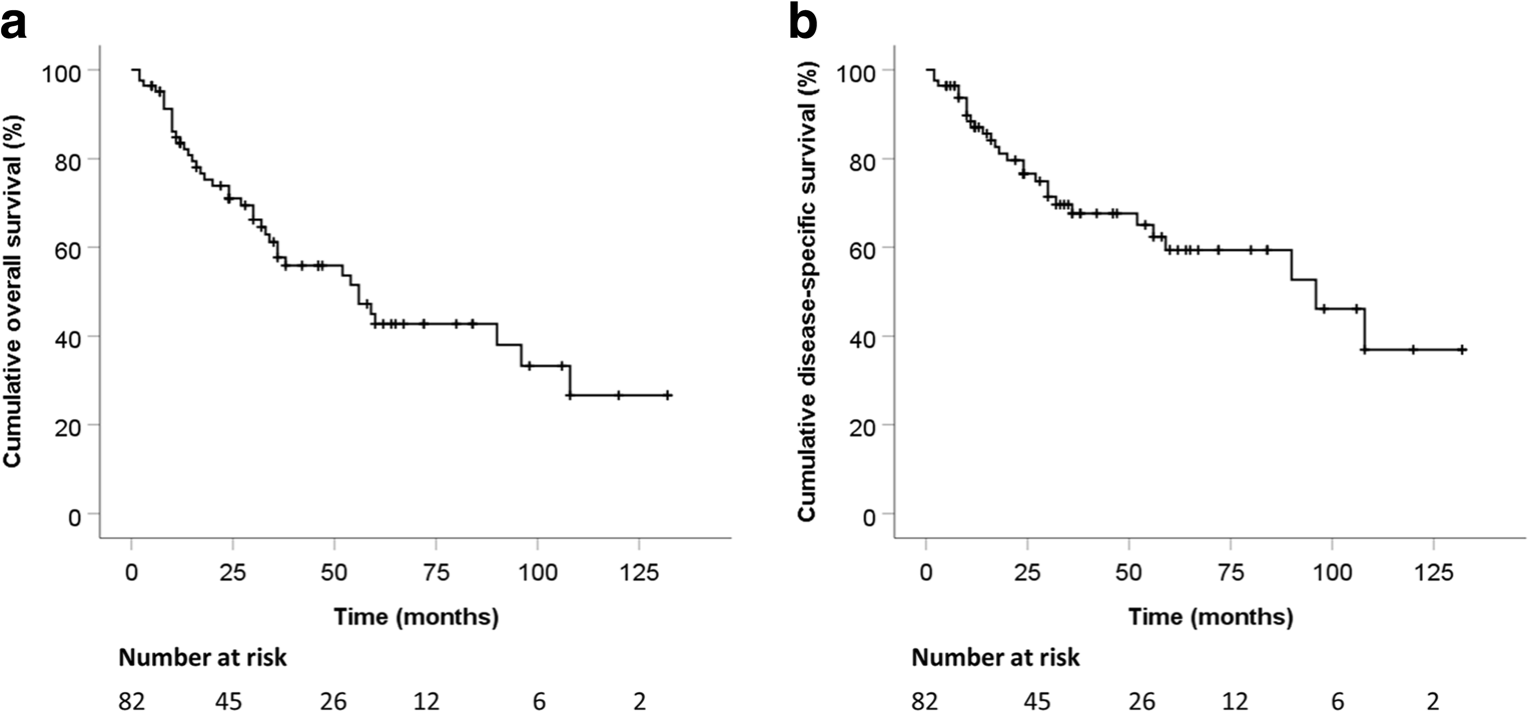
Overall (**a**) and disease-specific (**b**) survival of patients with cutaneous Merkel cell carcinoma

The 5-year OS and DSS were 52% and 66%, respectively (Fig. 1). The median OS and DSS for the whole series were of 62 months (SE 9.376, 95% CI 43.622–80.378) and 96 months (SE 21.371, 95% CI 54.112–137.888), respectively. Twenty-eight subjects (33% of the whole population) died of their disease (DOD).

**Fig. 2:**
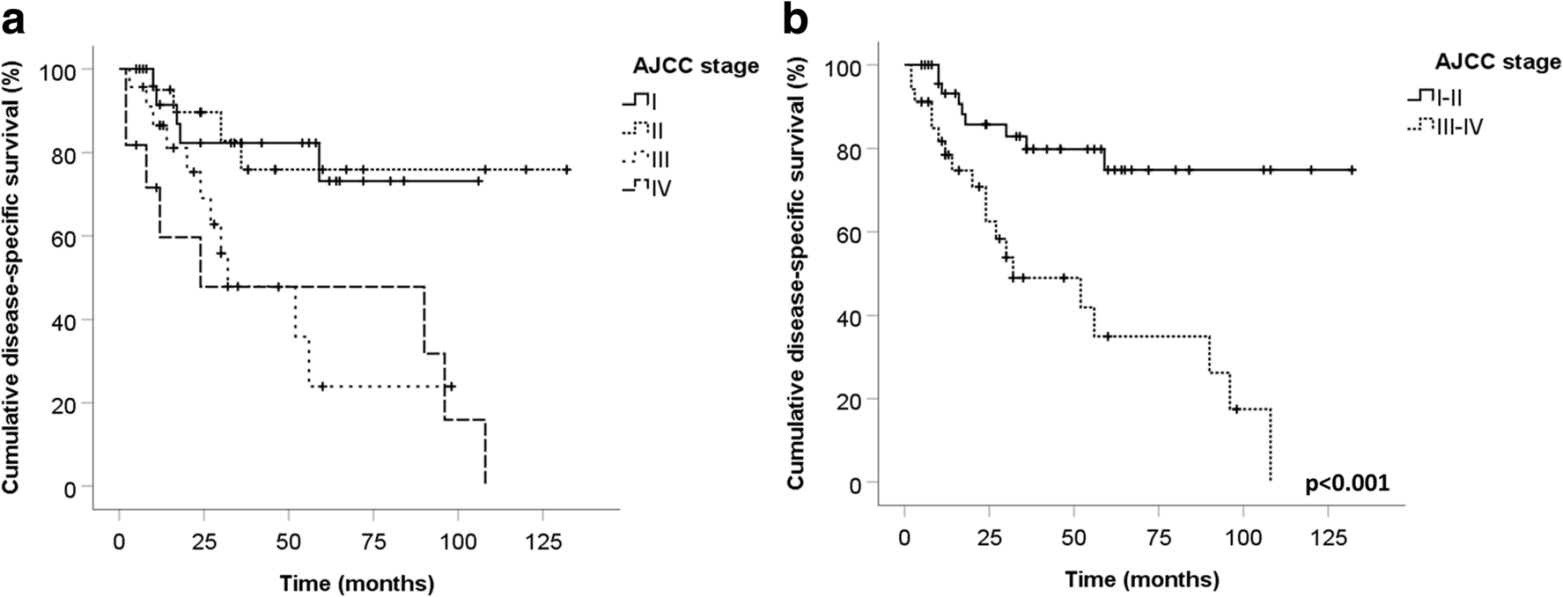
Disease-specific survival (DSS) related to AJCC tumor stage. Stages I and II presented similar DSS rates as compared to stages III and IV (**a**). Different DSS was observed when grouping stage I with II and compared with stage III with stage IV grouped together (**b**) (log-rank test *p* < 0.001)

**Fig. 3:**
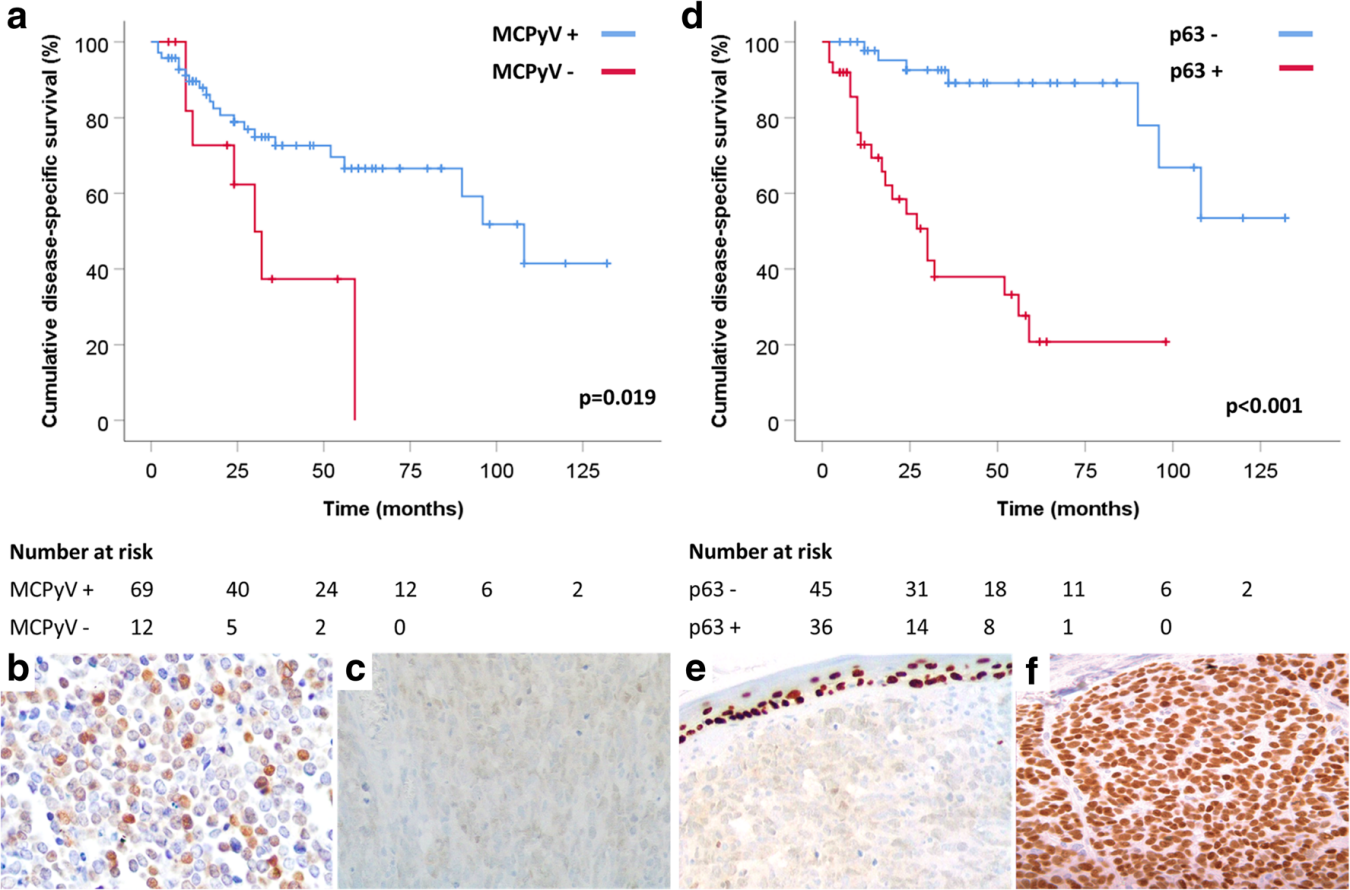
Patients with Merkel cell carcinomas expressing MCPyV show a better survival than patients with MCPyV-negative carcinomas (**a**). MCPyV immunoreactivity is nuclear (**b**). Image (**c**) is an example of a MCPyV-negative Merkel cell carcinoma. Expression of p63 by neoplastic cells is associated with a worse prognosis (**d**). In image (**e**), there is an example of p63-negative Merkel cell carcinoma. Note the internal control in the basal layer of the normal epidermis. p63 is expressed in the nuclei of cancerous cells (**f**)

In the whole group, the univariate analysis showed that AJCC stage (HR 1.864, 95% CI 1.295–2.682, *p* = 0.001), p63 immunohistochemical expression (HR 8.304, 95% CI 3.285–20.991, *p* < 0.001), absence of MCPyV immunoreactivity (HR 2.740, 95% CI 1.133–6.628, *p* = 0.025), and Ki67 index (HR 1.033, 95% CI 1.013–1.055, *p* = 0.002) were significantly associated with shorter DSS (Table 3). Early AJCC stages of disease (I and II) presented similar DSS rates. The most remarkable difference in survival analysis was observed grouping stages I and II together against grouped stages III and IV (log-rank test *p* < 0.001) (Fig. 2). Fifty-four percent of patients with MCPyV-negative MCCs died of disease versus 30% of patients with MCPyV-positive MCCs (*p* = 0.019). Seventy-six percent of patients with p63-positive MCCs died of disease, versus 24% of patients with p63-negative MCCs (*p* < 0.001) (Fig. 3).

**Table 3:** Cox univariate analysis of variables in relations to the disease-specific survival

Variable	HR (CI 95%)	*p* value
Age (log)	1.016 (0.978–1.055)	0.42
Sex (male)	2.05 (0.871–4.826)	0.1
Size (cm)	0.94 (0.75–1.178)	0.592
Site (head and neck)	1.766 (0.821–3.798)	0.145
AJCC stage (log)	1.864 (1.295–2.682)	0.001
Margin status (R1)	0.736 (0.296–1.833)	0.51
Tumor thickness (log)	1.015 (0.946–1.089)	0.676
Tumor growth (infiltrative)	1.371 (0.637–2.955)	0.42
Angioinvasion (present)	2.177 (0.921–5.145)	0.076
Mitosis × HPF (log)	0.996 (0.986–1.006)	0.466
p63 (expressed)	8.304 (3.285–20.991)	< 0.001
MCPyV (negative)	2.740 (1.133–6.628)	0.025
Ki67 index (log)	1.033 (1.013–1.055)	0.002

*HR*, hazard ratio; *MCPyV*, Merkel cell polyomavirus; *HPF*, high power field

**Fig. 4:**
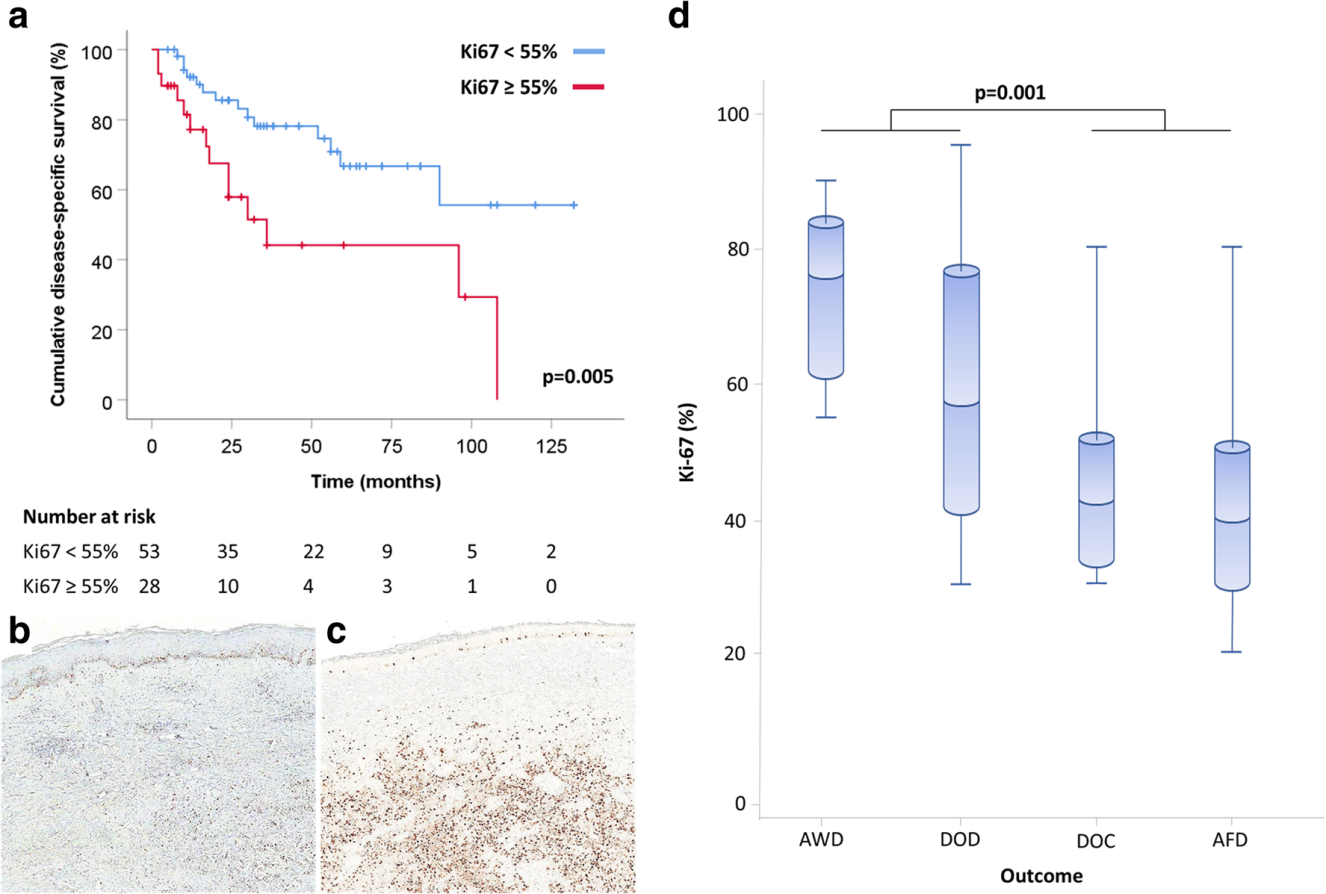
Kaplan-Meyer curve (**a**) demonstrating that patients with Merkel cell carcinomas showing a Ki67 proliferative index < 55% (**b**) show a better survival than patients with Merkel cell carcinomas with a Ki67 index > 55% (**c**). Merkel cell carcinomas with Ki67 index > 55% were observed more frequently in patients with disease, both died (DOD) and alive (AWD) than in patients alive free of disease (AFD) or died for other causes (DOC) (**d**)

Since Ki67 proliferative index > 55% has been demonstrated to be associated with worse prognosis in large series of digestive NECs [10, 11], we performed the Kaplan-Meyer estimator analysis using the same cut-off. Youden’s index for ROC curve analysis identified 50.5% and 57.5% Ki67 values as optimal cut-points to detect DOD patients (*J* = 0.214), corroborating the use of the threshold of 55%. Interestingly, when patients were classified according to outcome (alive and well, alive with disease, died of other cause, and died of disease), MCCs with Ki67 index > 55% were more frequently observed in patients with progressive/persistent disease (died of disease or alive with disease) than in patients free of disease (*p* = 0.001) (Fig. 4).

These four factors (stage, p63, MCPyV, Ki67) were entered into a multivariate analysis with patients’ age and gender considered as correction factors. p63 expression (HR 7.254, 95% CI 2.792–18.843, *p* < 0.001) and AJCC advanced stage (HR 1.934, 95% CI 1.239–3.018, *p* = 0.004) remained the only independent factors significantly affecting the DSS.

The prognostic role of the Ki67 proliferative index was also explored in the subgroup of MCPyV-positive tumors (71 cases). Similar to what observed in the analysis of the entire cohort, Ki67 proliferative index > 55% was statistically associated with DSS at the univariate Cox proportional-hazard regression analysis (*p* = 0.002, HR 1.041, 95% CI 1.0.15–1.067), but it was not an independent prognostic factor at the multivariate analysis as compared to p63 and AJCC advanced stage.

## Discussion

MCC of the skin is a rare neuroendocrine carcinoma with a reported 5-year overall survival (OS) ranging from 30 to 60% [12–14], and our findings are in line with this, showing a 5-year OS of 52%, worse than the 5-year DSS (66%). This probably reflects the characteristics of our cohort, where most of patients were elderly (mean age 76 years) with other comorbidities and fatal events for unrelated causes. A similar feature was observed by Jemec et al., whose series of MCCs showed an OS worse than DSS after a mean follow-up time of 68.2 months [15]. Several different clinico-pathological factors negatively influencing patients’ survival have been proposed for cutaneous MCC and included age, male gender, clinical evidence of lymph node metastasis, depth of tumor invasion, infiltrative growth pattern, loss of RB function, lack of MCPyV immunoreactivity, and p63 expression [16–19]. More recently, promoter methylation of the immune checkpoint receptor CD279 (PD-1) and intratumor lymphocyte subtypes have been suggested as additional prognostic factors [20, 21].

Ki67 proliferative index represents one of the most important biological markers routinely used as prognosticators in NENs. Its role was first demonstrated in pancreatic NETs [22, 23] and then in other digestive NETs [4] as well as in NETs of other sites (e.g., in the pituitary gland) [5, 6]. Recent data also suggest a prognostic role of Ki67 proliferative index in lung NENs and in non-epithelial NENs, like olfactory neuroblastoma [8, 24]. In addition to NETs, Ki67 proliferative index also plays a prognostic role in NECs, as observed in the digestive system; indeed, NECs with a Ki67 index > 55% show worse prognosis than NECs with Ki67 index < 55%, despite a better initial response of the former to chemotherapy [10, 11]. For all these reasons, the evaluation of Ki67 is a required (digestive system) or strongly recommended (other sites) parameter in the diagnostic work-up of NENs.

Despite the large available literature on the prognostic role of Ki67 index in NENs, the available data on MCC are heterogeneous, mainly due to the different methods used to assess Ki67 proliferative index and the relatively small number of cases studied in each series. Although some studies suggested an association between Ki67 index and a more aggressive clinical behavior [19, 25, 26], others did not confirm this data [15, 27–32] (Table 4). Although Ki67 is a continuous variable, the current stratification of patients is generally related to specific Ki67 cut-offs, which are able to separate patients in different prognostic categories. However, standardized method of evaluation such as that proposed by the WHO [4, 9] is mandatory. Since MCC may be considered as the cutaneous counterpart of visceral NECs and since no specific prognostic Ki67 cut-off has been identified for MCC, we decided to use the only available and efficient Ki67 threshold (55%), although it refers to digestive NECs [10, 11]. This choice was also supported by the Youden index analysis, which identified best cut-point Ki67 values at 52.5% and 57.5%. We observed an association between Ki67 proliferative index higher than 55% and tumor recurrence, in line with other published findings [27, 29, 35, 36, 39]. In addition, we demonstrated, at univariate analysis, the relationship between high Ki67 proliferative index and DSS, a finding not confirmed by previous studies (Table 4). Indeed, in three studies, only the association between Ki67 proliferative index and OS, but not DSS, was observed [19, 25, 26]. These discrepancies may be due to the different methods used to evaluate the Ki67 proliferative index and to the different cut-offs established for the survival analysis. Regarding the first point, we are the first to use the WHO recommendations by counting the number of positive cells in at least 500 tumor cells in hot spot areas on camera-captured printed images [4, 9]. As discussed above, the choice of 55% Ki67 cut-off was based on the analogy with digestive NECs and on the Youden index analysis. Our results on MCCs are in line with those observed in digestive NECs that showed different survivals, when separated in two groups based on Ki67 proliferative index (< 55% vs > 55%), [11]. However, although Ki67 proliferative index resulted significantly associated with prognosis at univariate analysis, together with tumor stage IV, MCPyV lack, and p63 expression [16–19], it was not an independent predictor at the multivariate analysis, where survival resulted independently influenced by p63 expression and tumor stage, only.

**Table 4:** Review of the literature: prognostic role of Ki67 in Merkel cell carcinoma

Reference	Year	Number of cases	Ki67 mean value	Ki67 cut-off	Correlation with recurrence and/or MTS	Correlation with DSS
Parrado [27]	1998	25	40%	nr	Yes	No
Carson [28]	1998	20	nr	nr	No^	No^
Jemec [15]	2000	13	nr	nr	nr	No
Erickson [33]	2003	39	36.8%	nr	nr	nr
Acebo [34]	2005	11	75%	nr	nr	nr
Fernandez-Figueras [35]	2005	24	46.67%	*	Yes	nr
Llombardt [29]	2005	20	nr	50%	Yes	No
Koljonen [36]	2006	24	47%	35%	Yes	nr
Tucci [30]	2006	12	nr	#	No	No
Belhocine [37]	2006	11	50%	nr	nr	nr
Pozo [38]	2007	27	46.97%	nr	nr	nr
Asioli [19]	2007	47	50.6%	65%	nr	nr°
Kim [31]	2008	19	nr	nr	nr	No
Sihto [16]	2011	91	nr	nr	nr	nr
Lim [32]	2012	95	60% (median)	50%	nr	No
Henderson [25]	2014	21	nr	nr	nr	No°°
Vujic [39]	2015	26	52%	§	Yes	nr
Iwasaki [26]	2015	28	54.5%	60%	nr	No°°°
Lezoux-Kozal [40]	2015	15	46%	nr	nr	nr
Orlova [41]	2018	32	nr	nr	nr	nr
Kitamura [42]	2018	10	53%	nr	nr	nr
Present series		84	51.3%	55%	Yes	No

*MTS*, metastases; *DSS*, disease-specific survival; *nr*, not reported; ^cases were considered positive when Ki67 > 10%; *not identified but metastases and recurrence were observed when Ki67 > 54%; ^#^not identified but worse prognosis were observed when Ki67 > 51.46%; °correlation with OS (*p*:0.001), but not in multivariate analysis; °°correlation with OS (*p*:0.0597); ^§^not identified but aggressive behavior was observed when Ki67 > 67%; °°°correlation with OS (*p*:0.048)

The prognostic role of p63 expression is in line with previous findings [19], and it may be related to its function as tumor suppressor as demonstrated in p63 knockout mice [43]. However, it is worth noting that different p63 isoforms exist and may have distinct effects on oncogenesis. Since the anti-p63 antibody used in this study does not distinguish among different isoforms, we are not able to identify the p63 isoform having the major pathogenetic role. Asioli et al. have demonstrated that TAp63a and DNp63a were the most frequently expressed isoforms (42%) in low-stage MCCs, suggesting that an early anomalous regulation of their expression might determine an aggressive phenotype [44].

In contrast to proliferation markers, a possible prognostic role of factors influencing apoptosis has been poorly investigated in MCCs. Indeed, although apoptotic cells were detected in MCCs using TUNEL, DNA ladder and immunohistochemistry for Fas (Apo-1/CD95) [45], the prognostic role of apoptotic index and of proteins and mediators involved in the apoptotic machinery (Bcl-2, p53, surviving, and CXCR4) remains to be elucidated [30].

In conclusion, this study, which integrates original findings with literature data, is the first one analyzing the prognostic role of Ki67 proliferative index in MCC using a standardized methods of Ki67 evaluation. Nevertheless, although Ki67 proliferative index was associated with recurrent disease and shorter patients’ survival at univariate analysis, it did not prove to independently influence prognosis, which in our relatively large series mainly depended on high tumor stage and p63 expression.
